# Characterization of *fliR*-deletion mutant Δ*fliR* from *Vibrio alginolyticus* and the evaluation as a live attenuated vaccine

**DOI:** 10.3389/fcimb.2023.1162299

**Published:** 2023-04-25

**Authors:** Fan Da, Xiaoju Wan, Guixiang Lin, Jichang Jian, Shuanghu Cai

**Affiliations:** Guangdong Provincial Key Laboratory of Aquatic Animal Disease Control and Healthy Culture, Shenzhen Institute of Guangdong Ocean University, Fisheries College of Guangdong Ocean University, Guangdong, China

**Keywords:** *Vibrio alginolyticus*, fliR gene, live attenuated vaccine, grouper, motility

## Abstract

*Vibrio alginolyticus* is the common pathogen affecting various species of marine organisms. It has been demonstrated that *fliR* is a necessary virulence factor to adhere and infect their hosts for pathogenic bacteria. Frequent disease outbreaks in aquaculture have highlighted the necessity of developing effective vaccines. In the present study, in order to investigate the function of *fliR* in *V.alginolyticus*, the *fliR* deletion mutant Δ*fliR* was constructed and its biological properties were evaluated, additionally, the differences in gene expression levels between wild-type and Δ*fliR* were analyzed by transcriptomics. Finally, Δ*fliR* was used as a live attenuated vaccine to immunize grouper via the intraperitoneal route to evaluate its protective effect. Results show that *fliR* gene of *V. alginolyticus* was identified as being 783 bp in length, encoding 260 amino acids, and showing significant similarity to homologs of other *Vibrio* species. The *fliR*-deletion mutant Δ*fliR* of *V. alginolyticus* was successfully constructed, and its biological phenotype analysis showed no significant differences in growth capacity and extracellular enzyme activity compared to the wild-type. However, a substantial reduction of motility ability was detected in Δ*fliR*. Transcriptomic analysis revealed that the absence of *fliR* gene is responsible for a significantly decreased expression of flagellar genes, including *flaA*, *flaB*, *fliS*, *flhB* and *fliM*. The *fliR*-deletion mainly affects the related pathways involved in cell motility, membrane transport, signal transduction, carbohydrate metabolism, and amino acid metabolism in *V. alginolyticus*. The efficacy of Δ*fliR* as a candidate of live attenuated vaccine were evaluated by intraperitoneal injection in grouper. The Δ*fliR* provided the RPS (Relative protection rate) of 67.2% against *V. alginolyticus* in groupers. The Δ*fliR* efficiently stimulated antibody production with specific IgM still detected at 42 d post-vaccination, and significantly elevated the activity of antioxidant enzymes like Catalase (CAT), Superoxide dismutase (SOD), and lactate dehydrogenase (LDH) in the serum. The higher expression levels of immune-related genes were observed in the immune tissues of inoculated grouper compared to the control. In conclusion, Δ*fliR* effectively improved the immunity of inoculated fish. The results suggest that Δ*fliR* is an effective live attenuated vaccine against vibriosis in in grouper.

## 1 Introduction

Intensive marine fish aquaculture is developing rapidly in coastal areas of China. With the fast expansion of mariculture, more and more diseases have outbroken and led to the huge economic loss. *Vibrio* is a kind of ubiquitous pathogens in marine and estuarine environments, and consumption of undercooked seafood or exposure to *Vibrio*-contaminated seawater may cause *Vibrio* infection (Adeleye et al., 2010; Mrityunjoy et al., 2013; Baker-Austin et al., 2018; Song et al., 2020). *Vibrio alginolyticus* is one of the dominant pathogens in the marine environment and associated with a variety of diseases in marine organisms such as fish, crustaceans, and mollusks (Mustapha et al., 2013; Li et al., 2021; Sadat et al., 2021; Yang et al., 2021). Investigations have demonstrated that *V. alginolyticus* is responsible for ulceration, bleeding, and black spots on the epidermis of various marine organisms, causing extremely bad effect to the aquaculture industry (Abdallah et al., 2011).

The pathogenic processes of *Vibrio* include adhesion, invasion and *in vivo* proliferation, acting through the production of pathogenic factors that interfere with the normal metabolism of host cells (Li et al., 2003). Bacterial adhesion to the host is an important step and the initial stage of bacterial infection (Qi et al., 2022). Motility is usually essential for bacterial adhesion. The flagellum is the primary organelle for the motility of many bacteria (Chaban et al., 2015). Bacterial flagella are large macromolecular complexes composed of a hook, filament, and basal body (Zhu et al., 2017). The filament is a helical propeller consisting of a repetitive protein monomer flagellin. The hook is a curved hollow cylinder attached to the basal body and is a flexible gimbal. The basal body is anchored to the cell membrane, houses the secretory apparatus, and provides the power for flagellar rotation (Mukherjee and Kearns, 2014). The flagellar biosynthetic protein *fliR* is an integral membrane protein thought to be located in the central pore of the MS ring of the bacterial flagellar type III protein exporter along with FlhA, FlhB, FliO, FliP and FliQ (Mukherjee and Kearns, 2014). Recent study have shown that *fliR* is required for severe damage to the intestinal epithelium in *Streptococcus mucilaginous* (Sina Rahme et al., 2022). Related study in *V. harveyi* have shown that FliA, FliR and FlrB are the essential proteins that mediate the bacterial adhesion (Qi et al., 2022). However, the role of *fliR* in *V.alginolyticus* has not been demonstrated.

Bacterial diseases in fish are considered a significant problem in aquaculture. Overuse of antibiotics causes the increasing bacterial resistance and drugs residue, and vaccines are emerging as an essential strategy to prevent fish diseases (Minor, 2015). Vaccines include inactivated vaccine, attenuated live vaccine, recombinant vaccine and DNA vaccine. Attenuated vaccines have many noteworthy advantages, including low stress on the fish, the possibility of soaking or oral vaccines, and the ability to induce effective humoral and cell-mediated immune responses (Mohd-Aris et al., 2019). Research on live attenuated vaccines dates back to measles, mumps, rubella, and varicella zoster (Goh et al., 2007; Dhillon and Curran, 2008; Schuster et al., 2008). Attenuated live vaccines for human diseases are among the most successful and cost-effective interventions in the history of medicine (Minor, 2015). Attenuated live vaccines are obtained by removing critical virulent genes from strongly virulent strain and weakening their virulence to form the avirulent strain. Research has indicated that adherence is an essential virulence characteristic of *V. alginolyticus* (Luo et al., 2016). In the present study, to understand the function of *fliR* in *V. alginolyticus*, we firstly constructed *fliR-*deletion mutant Δ*fliR* and studied its biological characteristics, then analyzed the differences in gene expression levels between the wild-type and Δ*fliR* by transcriptomics. Finally, Δ*fliR* was used as an attenuated live vaccine to immunize grouper *via* the intraperitoneal route and demonstrate its protective effect.

## 2 Materials and methods

### 2.1 Bacterial strains, plasmid, and experimental fish

Healthy pearl gentian grouper (weight: 50.0 ± 5.0 g) was purchased from a fish farm in Donghai Island (Zhanjiang). The experimental fish were cultured in a temporary pond for two weeks before being placed in a tank at 28°C with daily seawater changes. The fish were fed twice daily with a commercial diet. Before sampling, the experiment fish were anesthetized with MS222 (Sigma, USA), and the tissue (liver, spleen and kidney) was surgically sampled and stored in liquid nitrogen. *V. alginolyticus* HY9901 (wild-type) was isolated from the diseased grouper and conserved in our laboratory. The plasmid used to construct the mutant in this study was the PLP12CM suicide plasmid.

### 2.2 Construction of in-frame deletion mutant of *fliR* gene

*V. alginolyticus* HY9901 *fliR* gene was obtained from the whole bacterial genome assembly (GCA_023206775.1). In this study, in-frame deletion of the *fliR* gene was performed by overlapping extension PCR. The primers *fliR*-MF1/*fliR*-MR1 were used to amplify the upstream homologous arm fragment of *fliR* with a length of 763 bp. The primers *fliR*-MF2/*fliR*-MR2 were used to amplify the downstream homologous arm fragment of *fliR* with a length of 718 bp. Amplification procedure is following: 98°C for 3 min. 35 cycles of 98°C for 10 sec, 52/58°C for 20 sec and an elongation stage at 72°C for 1 min. The amplified *fliR* upstream and downstream homologous arm fragments were used as templates for overlapping PCR amplification. Amplification program is following: 98°C for 1 min followed by 35 cycles of 98°C for 10 sec, 68°C for 20 sec and an elongation stage at 72°C for 1 min. The fusion fragment was 1481 bp in length and was ligated to the suicide vector PLP12CM. The recombinant product was transformed into *Escherichia coli* β2163 receptor cells. The recombinant plasmids PLP12CM-*fliR* were transferred from *E. coli* β2163 into *V. alginolyticus* HY990 by conjugation. The single crossover mutants were screened on LB agar medium containing 20 μg/mL chloramphenicol and 0.3% D-glucose at 28°C. The second crossover mutants were selected on LB agar with 0.4% L-arabinose. The *V. alginolyticus fliR*-deletion mutants (Δ*fliR*) were confirmed by PCR with the primers pLP-UF/pLP-UR.

### 2.3 Characterization of Δ*fliR*

#### 2.3.1 Growth curve

The Δ*fliR* and wild-type strain were cultured in TSB liquid medium on a rotary shaker (120 rpm) in a 28°C incubator. The OD_600_ was measured by pipetting the bacterial solution every 2 h.

#### 2.3.2 Genetic stability

The Δ*fliR* was passaged 30 times continuously on the TSB medium, and colonies of each generation were amplified by PCR and sequenced to determine the genetic stability of Δ*fliR* for subsequent research.

#### 2.3.3 Measurement of extracellular protease activity

Sterile cellophane was tightly attached to TSA solid medium, and subsequently, wild-type and Δ*fliR* were coated on the cellophane separately. The solid medium was placed in a constant temperature incubator at 28°C for 24 h, followed by washing the cellophane with sterile phosphate buffered saline (PBS, pH 7.4), aspirating the suspension, and centrifuging at 5000 rpm for 30 min at 4°C. The supernatant obtained was filtered with a membrane (pore size 0.22 μm), 100 µl of the filtered liquid was added to 400 µl of 5 mg/mL azo casein solution, and the mixture was allowed to stand at 37°C for 30 min, then 400 µl of 10% trichloroacetic acid was added, and after standing for 30 min, sodium hydroxide was added. Finally, the mixture was read by spectrophotometer for absorbance at 442 nm.

#### 2.3.4 Motility assay

The Δ*fliR* and wild-type were inoculated with toothpicks on plates with an agar content of 0.3%. The plates were incubated overnight at 28°C to observe the size of swimming rings and measure the diameter of the swimming rings.

#### 2.3.5 Median lethal dose (LD50)

To investigate the pathogenicity of wild-type strains and deletion strains of the strain. The LD50 was determined. The wild-type and Δ*fliR* were incubated in TSB medium, respectively. After 12 h of incubation, the bacterial solutions were collected, and the concentrations of the two strains were adjusted to 10^4^, 10^5^, 10^6^, 10^7^ and 10^8^ CFU/mL with PBS. Healthy groupers were selected and divided into 2 groups (150 fish/group), each group was further divided into 5 subgroups, and each subset of fish was injected with different concentrations of bacterial solution in turn. The control group of 30 fish was injected with an equal volume of PBS. Mortality and morbidity monitoring was subsequently carried out for 2 weeks.

### 2.4 Transcriptomic analysis of wild-type and *fliR* mutant

The wild-type and Δ*fliR* were incubated in TSB medium with a speed of 100 rpm at 28°C. After 10 h of incubation, the bacterial solution was centrifuged at 6000 g for 15 min, and the sediment was dissolved with Trizol to extract RNA. RNA quality was assessed on an Agilent 2100 Bioanalyzer (Agilent Technologies, USA) and sequenced using the Illumina Novaseq6000 platform (China Gene Denovo Biotechnology Ltd.) for sequencing. Fastp (version 0.18.0) was used to filter the raw data. Mapping was performed regarding the GCA_001679745.1 genome. Three biological replicates were set for each sample. Transcriptomics data were commissioned for analysis by China Gene Denovo Biotechnology Ltd. Quantitative real-time PCR (qRT-PCR) is used to detect differential gene mRNA expression levels.

### 2.5 Dynamic distribution of *fliR* mutant in grouper tissues

Sixty groupers were divided randomly into 2 groups. The injection doses for the experimental and control groups were as described in 2.5. Three fish in each group were randomly selected at 1, 2, 3, 4, 6 and 8 d after injection and anesthetized with an excess of MS-222 (Sigma, USA), and liver, spleen and kidney tissues were collected. Each sample was weighed and grounded in 1 mL of sterile PBS. The suspensions were diluted 10, 100 and 1000 times of the homogenate and applied on TCBS agar plates to determine the number of bacteria. TCBS medium is a common method for isolating pathogenic Vibrio in medical clinics. In healthy fish tissue, there is barely the presence of *Vibrio*. The experiment was performed three times independently.

### 2.6 Immunization and challenge

Healthy groupers were divided randomly into 2 groups of 300 fish each, and the fish in each group were cultured separately in 10 buckets. The injection doses for the experimental and control groups were as above. After 6 weeks of immunization, each group of fish was divided into 2 subgroups, with subgroup 1 injected with 100 μl of 1 × 10^9^ CFU/mL wild-type and subgroup 2 injected with the same volume of PBS. Clinical signs and mortality of grouper were monitored for 2 weeks after challenge. RPS was calculated by the following formula: RPS (%) = [1-(Mortality of vaccinated fish/Mortality of control fish)] × 100%.

### 2.7 Serum IgM test

Tail vein blood was taken from the experimental and control groups at 1-7 weeks. Blood samples were left at room temperature for 1 h, centrifuged at 3000 rpm for 10 min at 4°C, and the supernatant (serum) was aspirated and stored at -80°C. For detection of specific IgM against *V. alginolyticus*, The serum was continuously diluted with 2-fold gradient (from 2^1^ to 2^12^ folds dilution). Specific IgM antibody levels were measured by ELISA at 1-6 weeks after vaccination. After 12 h of incubation, *V. alginolyticus* HY9901 was resuspended in PBS and adjusted the concentration to 1 × 10^8^ CFU/mL. The 100 µl of bacterial suspension was added to each well of a 96-well microtiter plate and incubated overnight at 4°C. The wells were washed with PBST three times and blocked using 100 μl of 5% non-fat powdered milk (Sangon Biotech, Guangzhou, China) for 2 h. And then, the wells were washed with PBST three times, then added to 2-fold gradient diluted grouper serum and incubated at 37°C for 1 h. The wells were washed with PBST three times, and added 100 μl 1:1000 mouse-anti-pearl gentian grouper IgM polyclonal antibody as first antibody and incubated at 37°C for 1 h. The wells were washed with PBST three times, and incubated 100 μl 1:2000 horseradish peroxidase (HRP)-conjugated goat anti-mouse IgM as the secondary antibody, and incubated for 3 h at room temperature. After washing three times with PBST, color development buffer was added, and the absorbance was read at 450 nm with a spectrophotometer after 20 min of incubation. The experiment was performed three times.

### 2.8 Enzyme activity in fish serum

Superoxide dismutase (SOD) is involved in the body’s natural immunity. catalase (CAT) maintains the redox balance of the immune system. lactate dehydrogenase (LDH) rises in the serum when the tissues of an organism are damaged. The activity of these three enzymes was therefore measured after immunization. SOD, LDH, and CAT concentrations were measured using commercial kits from Nanjing Chengjian Institute of Biological Engineering and according to the manufacturer’s instructions. All samples were measured on a microplate reader. The experiment was performed three times.

### 2.9 Expression analysis of immune-related genes

The liver, spleen and kidney of fish in experimental and control groups were collected for qRT-PCR on days 0, 1, 2, 3, 4, 5 and 6 after inoculation. The qRT-PCR was performed to detect the expression levels of *caspase8*, *CD4*, *IgM*, *MHC-IIβ*, *IL6* and *TNF-α*. The genes selected are grouper innate and adaptive immunity-related genes. Primers are shown in **Table 1**, and *β-actin* was used as an internal reference. The qRT-PCR procedure was 10 min at 95°C. 40 amplification cycles of denaturation at 95°C for 30 s, annealing at 55°C for 30 s and extension at 72°C for 30 s. The expression levels of the related immune genes were analyzed by the 2^-ΔΔCT^ method. The experiment was performed three times.

**Table 1:** Primers used for the PCR of this experiment.

Concentration (CFU/mL	HY9901	Death rate (%)	Δ*fliR*	Death rate (%)	Control (PBS)	Death rate (%)
10^8^	30	86%	30	65%	0	–
107	30	80%	30	47%	0	–
10^6^	30	66%	30	5%	0	–
10^5^	30	26%	30	–	0	–
10^4^	30	23%	30	–	0	–
0	30	–	30	–	30	0

### 2.10 Statistical analysis

All statistical analyses were performed using one-way ANOVA, and all data are expressed as mean ± standard error. Statistical significance (P < 0.05) differences between groups were tested by Duncan’s multiple range test.

## 3 Results

### 3.1 Sequence analysis of *fliR* and construction of *fliR* mutant

**Figure 1:**
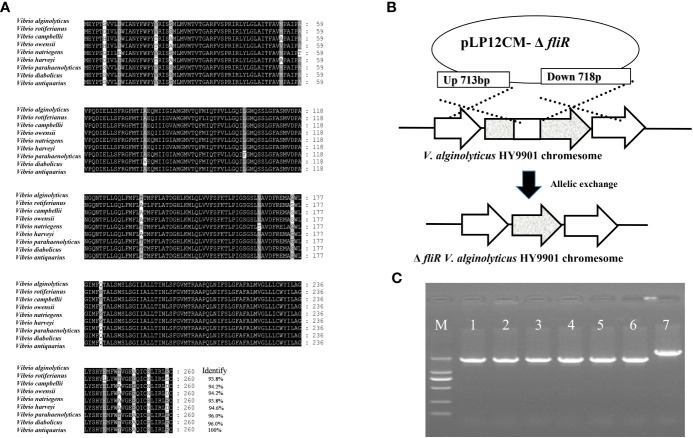
Identification of *fliR* gene from *V. alginolyticus* HY9901 strain and construction of *fliR* mutant strain. Multiple alignments of the *fliR* amino acid sequences of *V. alginolyticus* HY9901 with other Vibrio species **(A)**. The *fliR* gene of *V. alginolyticus* HY9901 was knocked out by allele exchange **(B)**. PCR amplification of wild-type *V. alginolyticus* HY9901 (lane 6), *fliR* mutant strain (lanes 1-5). Lane M is a 1 kb ladder. The PCR product of the mutant strain is smaller than that of wild-type *V. alginolyticus* HY9901 due to the partial deletion of the corresponding gene. The construction of the deletion strain was confirmed by PCR amplification **(C)**.

The *fliR* gene was identified as being 783 bp in length, encoding 260 amino acids, and multiple sequence comparisons showed significant similarity to homologs of other *Vibrio* species (**Figure 1A**). To generate the *fliR*-deletion mutation Δ*fliR*, the intermediate region of the *fliR* gene was deleted by allelic exchange (**Figure 1B**). The deletion of 717 bp of *fliR* gene truncation by allele exchange was confirmed by PCR amplification and subsequent sequencing (**Figure 1C**). The sequence of the fliR has been uploaded to the GenBank database with accession number OQ813764.

### 3.2 Characterization of *fliR* mutant

**Figure 2:**
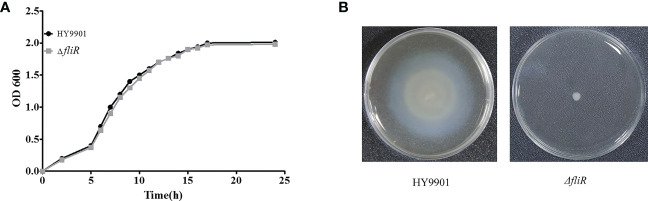
The biological properties of *V. alginolyticus* HY9901 compared with the *fliR* mutant strain. HY9901 and mutant strain growth curves **(A)**, values of OD600 of the bacterial suspensions were measured at different time points. Motility measurements. The swarming diameter of the wild-type strain was about 35 mm, and that of the mutant strain was 3 mm **(B)**.

The Δ*fliR* had stable genetics after 30 consecutive passages on TSB medium and its identification by PCR. Characterization of the biological phenotype of Δ*fliR* demonstrated that the deletion of *fliR* failed to significantly affect the growth ability and extracellular protease activity of *V. alginolyticus* (**Figure 2A**). However, after 12 h of incubation, the motility assay revealed that the swarming diameter of the wild-type was about 35 mm, and the swarming diameter of Δ*fliR* was only 3 mm, indicating that *fliR*-deletion had a significant effect on the motility of *V. alginolyticus* (**Figure 2B**). The LD_50_ of the wild-type was found to be 2.37×10^5^ CFU/mL, while the LD_50_ of Δ*fliR* was 3.48×10^7^ CFU/mL (**Table 2**), indicating that the deletion of *fliR* significantly diminished the pathogenicity of *alginolyticus* (**Table 3**).

**Table 2:** Experiment of LD50.

Primers	Primer sequences (5′-3′)	Amplification target
*fliR*-MF1	GGAATCTAGACCTTGAGTCGTGAACGAAATCAACGACAAAGC	Obtaining a fragment of the upstream homologous arm of fliR.
*fliR*-MR1	TCCAAGCGAATAAGCCGACATGCGATCCAATCTAAGACAACG	
*fliR*-MF2	CGTTGTCTTAGATTGGATCGCATGTCGGCTTATTCGCTTGGA	Obtaining fliR downstream homologous arm fragments.
*fliR*-MR2	ACAGCTAGCGACGATATGTCCGTCTTTGACTTCTTGCTTGGT	
pLP-UF	GACACAGTTGTAACTGGTCCA	Screening for recombinant clones with upstream and downstream homologous arm insertions.
pLP-UR	CAGGAACACTTAACGGCTGAC	
*fliR* -TF	TTCTGATGACCTCGTTCACTCG	Validation of deletion mutant clones.
*fliR* -TR	GCTTGAGGAACATCCGCCAT	
EpCaspase-8-qF	TGCTTCTTGTGTCGTGATGTTG	Real time PCR of Caspase-8.
EpCaspase-8-qR	GCGTCGGTCTCTTCTGGTTG	
EpCD4-qF	TTGCGGTGCAAAATCCACTG	Real time PCR of CD4.
EpCD4-qR	TGCCATCAGTCCAGGACAAC	
EpMHCIIβ-qF	GCCGCCACGCTACAGGTTTCTA	Real time PCR of MHCIIβ.
EpMHCIIβ-qR	TCCATCGTGGTTGGGGATGATC	
EpIgM-qF	TACAGCCTCTGGATTAGACATTAG	Real time PCR of IgM.
EpIgM-qR	CTGCTGTCTGCTGTTGTCTGTGGAG	
EpIL6-qF	GCATGTGCTTAAAGTATCCTGGTC	Real time PCR of IL6.
EpIL6-qR	TGCAAATTGTGGTCGGTATCTC	
EpTNFα-qF	TAGAACAACCCAGCAAAC	Real time PCR of TNFα.
EpTNFα-qR	ACCAGCGGTAAAGGCAAC	
Epβ-actin -qF	GGACAGCTACGTTGGTGATGA	Real time PCR of β-actin.
Epβ-actin -qR	TGGTCACAATACCGTGCTCAATG	

**Table 3:** The biological properties of *Vibrio alginolyticus* HY9901 compared with the *fliR* mutant strain.

Characteristics	HY9901	Δ*fliR*
Activity of ECPase(A422)	1.25 ± 0.3	1.08 ± 0.3
Swarming(mm)	35.32	3.64
LD50(CFU/mL)	2.37x10^5^	3.48x10^7^

### 3.3 Global transcriptional profile of *ΔfliR*

**Figure 3:**
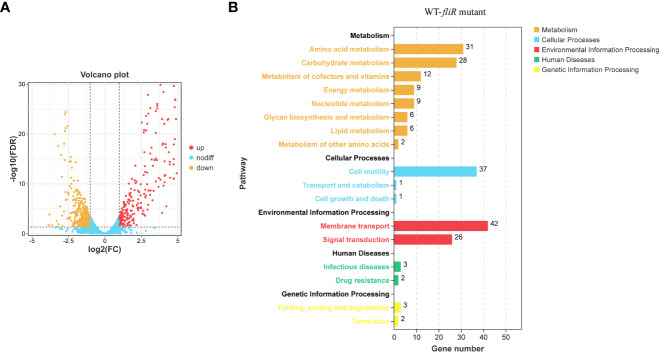
Comparison of the transcriptomes of *fliR* mutant strains and wild-type strains. The transcriptomes of the wild and mutant strains identified the number of differentially expressed genes, with 280 up-regulated genes and 398 down-regulated genes in the mutant strains **(A)**. The Kyoto Encyclopedia of Genes and Genomes (KEGG) enrichment analysis shows that mutations in *fliR* mainly affect pathways related to carbohydrate metabolism, amino acid metabolism, cell motility, membrane transport, and signal transduction **(B)**.

To investigate the function of *fliR* in *V. alginolyticus*, RNA-seq was used to investigate the related pathways affected because of the absence of *fliR* gene. The transcriptome of wild-type and Δ*fliR* was compared to identify differentially expressed genes (DEGs). Gene expression data were normalized in all replicates for differential expression analysis. To analyze the DEG, we chose a false discovery rate of less than 5% and a fold change >1.5 to obtain significant differences (All data are analyzed through the online platform of China Gene Denovo Biotechnology Ltd). Several differentially expressed genes were randomly selected for qPCR to validate transcriptome results (not shown in the article). The results showed that a total of 678 genes were found to be differentially expressed. Among them, 398 genes were downregulated, and 280 genes were upregulated in Δ*fliR* compared to wild-tpye (**Figure 3A**). Kyoto Encyclopedia of Genes and Genomes (KEGG) enrichment analysis revealed that a large number of DEGs were mainly associated with carbohydrate metabolism, amino acid metabolism, cell motility, membrane transport, signal transduction (**Figure 3B**). Most of these significantly down-regulated genes were associated with flagella (**Table 4**). Raw transcriptome sequencing data were submitted to GenBank with the accession number PRJNA954769.

**Table 4:** Motility-related genes were down-regulated (>1.5-fold change) in the mutant strains.

Cluster	Gene	log2(fc)	Symbol	Function
1	VAGM002496	-0.89915	flgB	Flagellar basal-body rod protein FlgB [Vibrio alginolyticus|Vibrio sp.]
	VAGM002497	-0.985	flgC	Flagellar basal body rod protein FlgC [Vibrio|Vibrio alginolyticus|Vibrio harveyi|Vibrio sp.]
	VAGM002498	-0.56764	–	Flagellar basal body rod modification protein FlgD [Vibrio|Vibrio alginolyticus|Vibrio sp.]
	VAGM002499	-0.8092	–	Flagellar hook protein FlgE [Vibrio|Vibrio alginolyticus|Vibrio sp.]
	VAGM002500	-1.58313	–	Flagellar basal body rod protein FlgF [Vibrio|Vibrio alginolyticus|Vibrio sp.]
	VAGM002501	-1.60902	flgG	Flagellar basal-body rod protein FlgG [Vibrio|Vibrio alginolyticus|Vibrio natriegens|Vibrio parahaemolyticus|Vibrio sp.]
	VAGM002502	-1.75332	flgH1	Flagellar L-ring protein FlgH [Vibrio|Vibrio sp.]
	VAGM002503	-0.93758	flgI2	Flagellar P-ring protein FlgI [Vibrio|Vibrio alginolyticus]
	VAGM002505	-1.82675	–	Fagellar hook-associated protein FlgK [Vibrio alginolyticus]
	VAGM002506	-2.24823	–	Flagellar hook-associated protein FlgL [Vibrio sp. 712i1]
2				
	VAGM003930	-0.29016	fliQ	Flagellar biosynthesis protein FliQ [Vibrio|Vibrio alginolyticus|Vibrio sp.]
	VAGM003931	-0.80537	fliP	Flagellar biosynthesis protein P [Vibrio alginolyticus]
	VAGM003932	-0.7989	fliO	Flagellar biosynthesis protein FliO [Vibrio|Vibrio sp.]
	VAGM003934	-0.2011	fliM	Flagellar motor switch protein FliM [Vibrio alginolyticus|Vibrio harveyi|Vibrio sp.]
	VAGM003936	-0.45234	–	Flagellar hook-length control protein FliK [Vibrio sp. OY15]
3	VAGM003949	-1.86758	fliS	Flagellar biosynthesis protein FliS [Vibrio|Vibrio alginolyticus|Vibrio sp.]
	VAGM003950	-2.6782	–	Flagellar rod protein FlaI [Vibrio|Vibrio sp.]
	VAGM003951	-2.67397	fliDP	Flagellar hook-associated protein 2 [Vibrio alginolyticus 40B]

### 3.4 Dynamic distribution of *ΔfliR*

**Figure 4:**
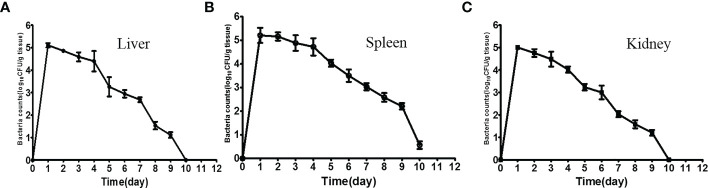
Dynamic distribution of *fliR* Mutant strain. Bacterial loads in the liver **(A)**, spleen **(B)**, and kidney **(C)** of experimental fish 12 h after intraperitoneal injection.

Following intraperitoneal injection, tissue samples from experimental fish were sampled consecutively for the bacterial load. After 12 h of administration, the maximum numbers of Δ*fliR* in the liver (**Figure 4A**), spleen (**Figure 4B**), and kidney (**Figure 4C**) were found to be about 10^6^ CFU/g. Bacterial counts were found to diminish in all tissues tested 1 day after inoculation, and a significant decline in counts was detected after 5 days, while only a slight amount of Δ*fliR* were isolated in the spleen after day 10 after inoculation, while no Δ*fliR* were detected in the kidney and liver.

### 3.5 Immunoprotective effect of *ΔfliR* in groupers

**Figure 5:**
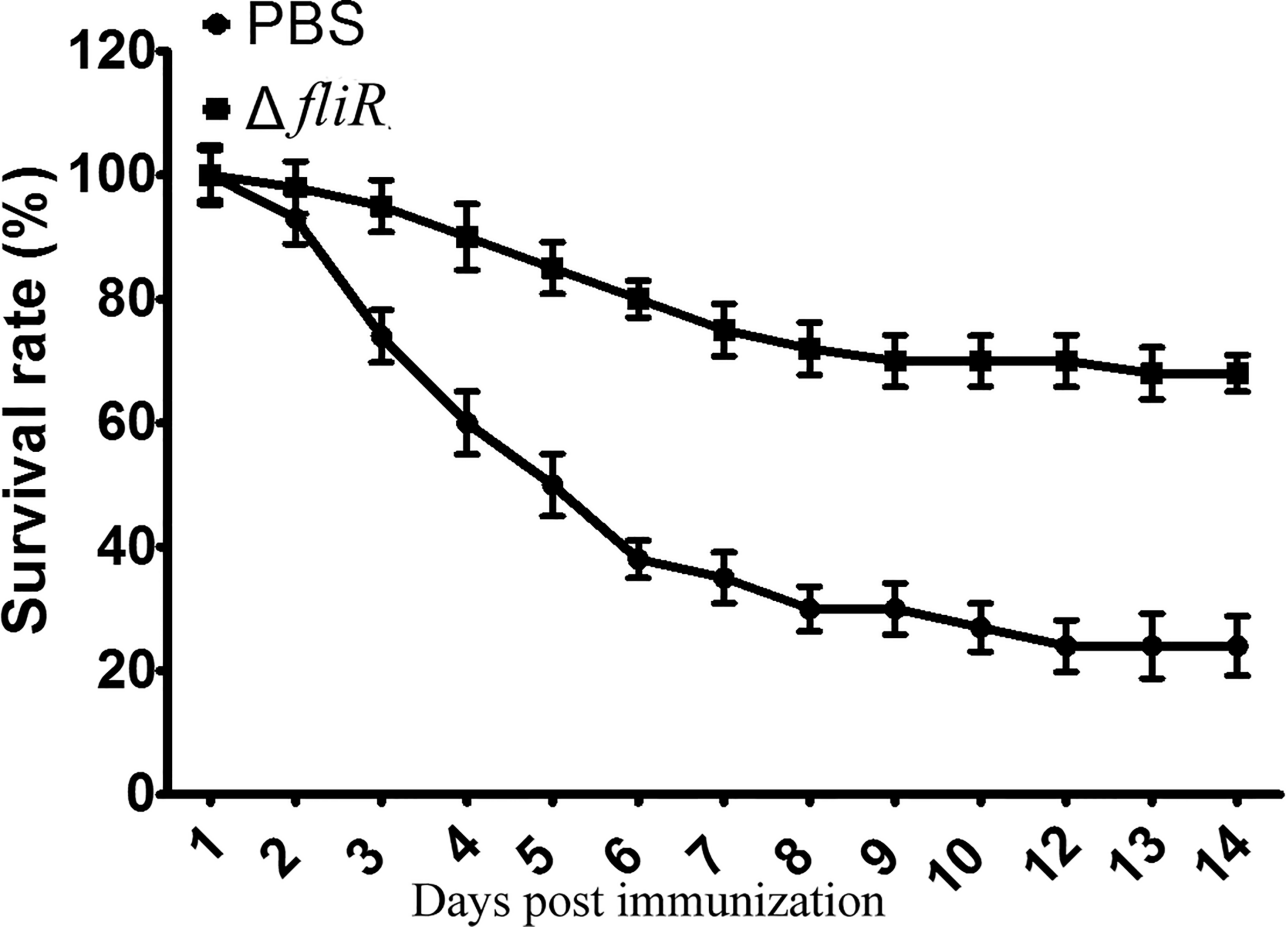
Determination of the immune protection rate of the *fliR* mutant strain against grouper. After infection with the wild-type strain, the control group had a significantly higher mortality rate three days after injection than the immunized group, with 68.45% and 24.54% mortality in the control and immunized groups, respectively.

After 4 weeks of vaccination, groupers from the immunized and control groups were challenged with wild-type. It was demonstrated that after stimulation with wild-type, mortality was observed in both the control and vaccine groups. In contrast to the immunized group, the control group showed a significantly higher number of mortalities during 3-7 days post-challenge, and the dead grouper exhibited terminal congestion of the caudal, ventral, and dorsal fins and ulcerated lesions with variable sites on the body surface. At 2 weeks post-challenge, the mortality rates were 68.45% and 24.54% in the control and immunized groups, respectively, and the RPS was calculated to be 67.2% (**Figure 5**).

### 3.6 Specific antibody titers after immunization of *ΔfliR* against grouper

**Figure 6:**
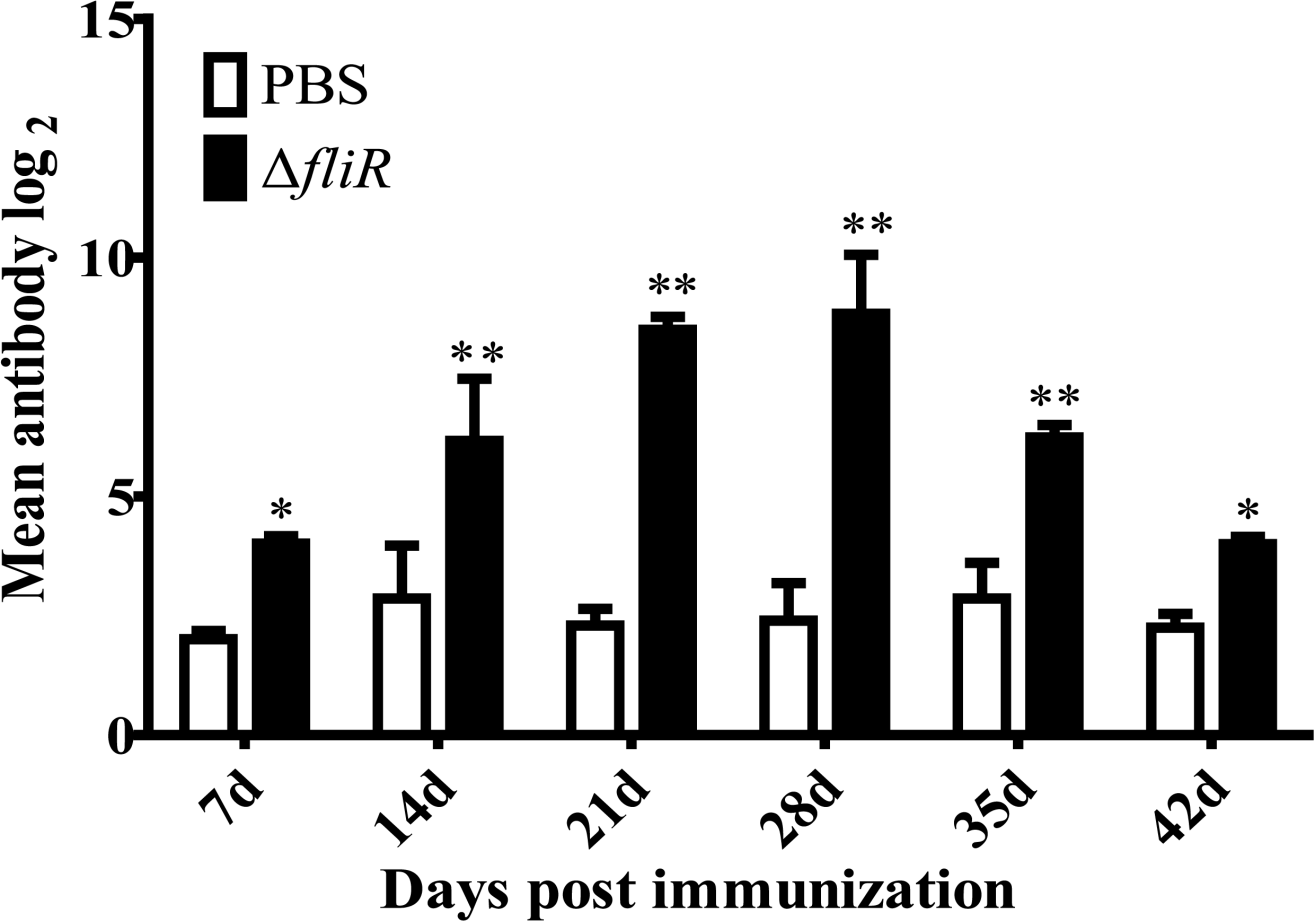
Determination of specific antibody titers following immunization of grouper with the *fliR* mutant strain. ELISA analyzed the IgM antibody titers in experimental fish 1-6 weeks after administration of the mutant strain. A consistent increase in serum antibody titers was seen in the experimental group from days 1-28, reaching a peak level on day 28, with a subsequent decrease. *indicate significant differences (p<0.05), **indicate extremely significant differences (p<0.01).

The ELISA analyzed the antibody titers of IgM in experimental fish from 1-6 weeks post-vaccination. The results indicated that the specific antibody titers of goupers in experimental group were significantly higher than those in the control group. A trend of continuous increase was observed in the experimental group from 1-4 weeks, with the highest level reached at 4th week, followed by a decrease in titers detected from 5th week (**Figure 6**).

### 3.7 Enzyme activity assay

**Figure 7:**
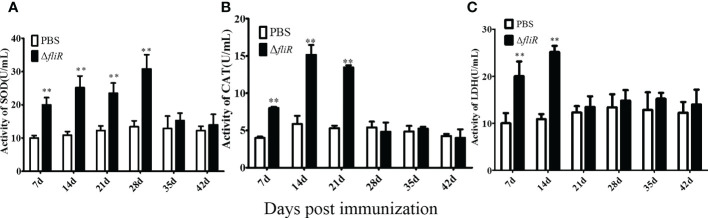
Determination of the enzymatic activities of SOD **(A)**, CAT **(B)**, and LZM **(C)** in fish serum. SOD activity peaked at week four and returned to normal by week 5. CAT and LDH activity peaked at week two and returned to normal by week 3. *indicate significant differences (p<0.05), **indicate extremely significant differences (p<0.01).

The enzyme activities of the groupers were measured in experimental and cotrol groups. Compared to that in the control, SOD activity increased significantly during 1 to 4 weeks post-vaccination, reached its highest value at 4th week, and returned to normal levels from 5th week. CAT activity increased significantly and reached its peak at 2nd week, and then decreased from 3rd week. LDH activity increased significantly and peaked at 2nd week, and returned to normal levels from 3rd week. These results suggest that Δ*fliR* activates the activity of antioxidant enzymes in the serum of inoculated fish (**Figure 7**).

### 3.8 qRT-PCR analysis of the expression of immune-related genes

**Figure 8:**
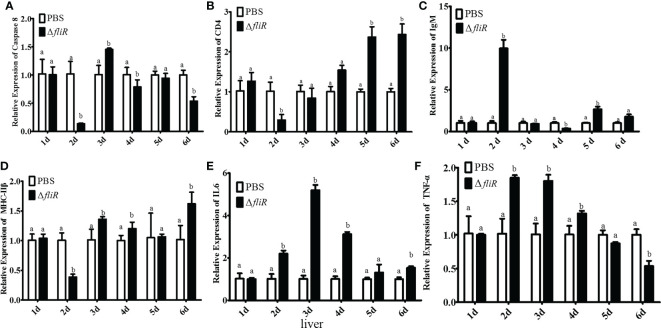
The expression levels of immune-related genes in grouper liver after immunization were detected by qRT-PCR for **(A)** *Caspase8*, **(B)** *CD4*, **(C)** *IgM*, **(D)** *MHC-IIβ*, **(E)** *IL6*, **(F)** *TNF-α*. Groups with notable differences are labeled with different letters above the bars. *β-actin* was chosen as the reference gene. Data are shown as mean ± SEM (n = 3).

**Figure 9:**
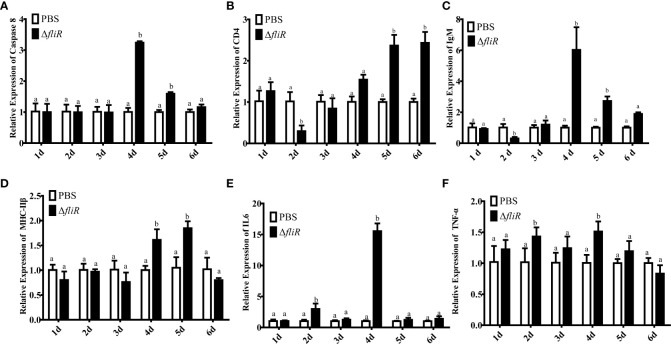
The expression levels of immune-related genes in grouper spleen after immunization were detected by qRT-PCR for **(A)** *Caspase8*, **(B)** *CD4*, **(C)** *IgM*, **(D)** *MHC-IIβ*, **(E)** *IL6*, **(F)** *TNF-α*. Groups with notable differences are labeled with different letters above the bars. *β-actin* was chosen as the reference gene. Data are shown as mean ± SEM (n = 3).

**Figure 10:**
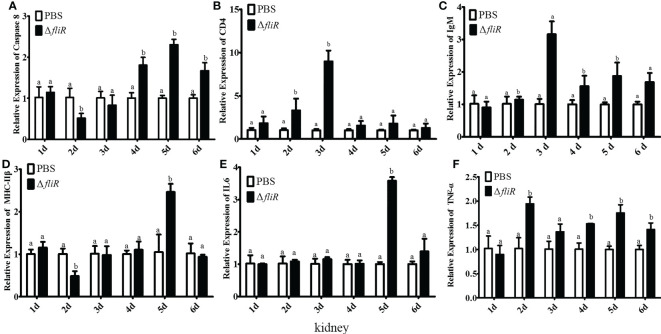
The expression levels of immune-related genes in grouper kidney after immunization were detected by qRT-PCR for **(A)** *Caspase8*, **(B)** *CD4*, **(C)** *IgM*, **(D)** *MHC-IIβ*, **(E)** *IL6*, **(F)** *TNF-α*. Groups with notable differences are labeled with different letters above the bars. *β-actin* was chosen as the reference gene. Data are shown as mean ± SEM (n = 3).

The immune response to the immune-related genes *caspase8*, *CD4*, *IL6*, *IgM*, *MHC-IIβ* and *TNF-α* in the liver (**Figure 8**), spleen (**Figure 9**), and kidney (**Figure 10**) of grouper was assessed by qRT-PCR after immunization. The results demonstrated that *caspase8* expression levels were significantly elevated at 3rd and 4th day in the liver, and at 4th day in the spleen. *CD4* and *IgM* expression levels were dramatically elevated and reached peaking value at 2nd day in the liver and kidney, and significantly increased in the spleen at 5th and 6th day. *MHC-IIβ* expression levels increased considerably in the liver at 3rd, 4th and 6th day, in the spleen at 4th and 5th day, and in the kidney at 5th day. *IL6* expression levels notably increased with the highest value appearing at 3rd day in the liver, at 4th day in the spleen, and at 5th day in the kidney. *TNF-α* expression levels increased remarkably from 2nd day in all 3 tissues, and decreased at 5th day in the liver and spleen. These results suggest that Δ*fliR* stimulates the expression of innate and adaptive immune-related genes in inoculated fish.

## 4 Discussion

Bacteria control the flagella for motility, enabling them to swim toward a favorable environment (Xie et al., 2011). Pathobacteria adhere to the surface of the host using flagella during the initial stages of infection, and consequently, flagella contribute essentially to the pathogenicity of bacteria (Li et al., 2003). Assembly of the flagellum is facilitated by its intrinsic protein export apparatus, the type III secretion system (T3SS) in pathogenic bacteria (Minamino et al., 2019). A series of genes regulate bacterial flagella’s number, location, and biosynthesis. *V. alginolyticus* forms a single flagellum at the polar end of the cell, and FlhFG regulates flagellum formation and number. Overexpression of FlhF in *V. alginolyticus* contributed to a multipolar flagellar phenotype, while mutation of *flhF* gene caused a flagellar-free phenotype. The function of FlhG is reversed from that of FlhF (Homma et al., 2022). The absence of FlhA, FlhB, FliI or FliR restrained the twitching motility of *Lysobacter enzymogenes*, implicating that FlhA, FlhB, FliI and FliR could be involved in regulating the function of twitch motility (Fulano et al., 2020). Silencing of FliA, FliR, and FlrB in *V. harveyi* significantly reduced bacterial adhesion, biofilm formation, motility, and flagellar synthesis (Qi et al., 2022). Investigation of *S. mucilaginous* infection of *Drosophila melanogaster* showed *fliR* as a virulence factor involved in *S. marcescens* mediated destruction of intestinal epithelial cells, which ultimately leads to the death of the host (Sina Rahme et al., 2022). Thus, the flagellum is regulated by a range of flagellum-associated genes, and the flagellum genes are involved in pathogenicity to the host.

It has been shown that FliR combined with FliP and FliQ forms the transmembrane output gate complex of the type III flagellin output apparatus and is associated with the injector of T3SS secretion system (Minamino et al., 2019). Scanning under electron cryomicroscopy showed that the core complex contains a single copy of FliR. The FliO ring complex protects FliP against proteolytic degradation and promotes the formation of the stable FliP5FliR1 complex. FlhA and FlhB are associated with the FliR/FliP/FliQ core complex (Minamino et al., 2019). Elimination of *csrA* gene in *H. pylori* J99 exhibited the loss of motility and reduced adherence, and transcription of mRNAs of FlaA and FlaB in the mutant strain was reduced to only 40% and 16%, respectively (Kao et al., 2014). After the knockout of FliS in *Campylobacter jejuni*, mutant strain behaved non-motile, showed reduced levels of FlaA/B and FlaC flagellin, and carried severely truncated flagella (Radomska et al., 2017). In *Listeria monocytogenes*, after deletion of *flhB*, *fliM* or *fliY* gene, the transcript levels of flagella-related genes *flaA*, *fliM*, *fliY*, *lmo0695*, *lmo0698*, *fliI* and *fliS* were significantly down-regulated, and bacterial motility and flagellar synthesis was utterly abolished (Cheng et al., 2018). Taken together, *fliR* has an essential function in the pathogenicity of pathogenic bacteria, however, the role of *fliR* on *V. alginolyticus* has not been studied.

The present study demonstrated that the absence of *fliR* resulted in a significant reduction of mRNAs for *flaA*, *flaB*, *fliS*, *flhB* and *fliM*. Mutation of *fliR*-deletion mainly affected pathways related to cell motility, membrane transport, signal transduction, carbohydrate metabolism, and amino acid metabolism in *V. alginolyticus*. Hence we observed that the swarming diameter of the *fliR* mutant strain was significantly smaller compared to the wild strain, suggesting that the absence of *fliR* resulted in reduced motility of Vibrio alginolyticus.

Aquaculture has become an economically important agribusiness worldwide. The primary obstacle to aquaculture development is the infectious diseases that cause severe economic losses. Live attenuated vaccines offer advantages against diseases caused by some pathogens in mariculture fish. Deletion or mutation of essential virulence-related genes of wild-type to make them weakly virulent strains (Mohd-Aris et al., 2019). The mutant do not cause fish mortality after vaccination and can exist in fish for a long time, effectively stimulating the host to produce antibodies and high expression of immune genes (Minor, 2015).

The experiments of bacterial dynamic distribution and ELISA analysis showed that *fliR* mutant survived for 10 days in the immune tissues of the inoculated fish, and efficiently stimulated IgM antibody production. SOD is a natural metalloenzyme used to scavenge large amounts of reactive oxygen radicals generated by the body’s redox reactions to maintain the integrity of the cellular structure, genetic material, and to participate in the body’s natural immunity (Garcia et al., 2017). Organisms can eliminate pathogenic bacteria through reactive oxygen species. CAT abolishes excess reactive oxygen species to avoid cell damage and maintain the redox balance of the immune system (Wang et al., 2013). LDH is a glycolytic enzyme and ascends in the serum when the organism’s tissues are damaged (Ding et al., 2017). In this study, the activities of CAT, SOD and LDH in grouper serum were significantly increased after the administration of *fliR* mutant. Furthermore, Δ*fliR* significantly increased the innate and adaptive (humoral and cellular) immune-related gene expression levels, and effectively promoted the survival rate of groupers.

In conclusion, we have constructed *V. alginolyticus fliR*-deletion mutant Δ*fliR* and analyzed the biological phenotype and transcriptome of the mutant. The protective effect of Δ*fliR* as a live attenuated vaccine against vibriosis in groupers was evaluated. The Δ*fliR* effectively stimulated the humoral and cellular immunity of the inoculated fish. The results indicate that Δ*fliR* is an effective live attenuated vaccine against vibriosis in cultured grouper.

## Data availability statement

Raw transcriptome sequencing data were submitted to GenBank with the accession number PRJNA954769. The sequence of the fliR has been uploaded to the GenBank database with accession number OQ813764.

## Ethics statement

The animal study was reviewed and approved by Guangdong Provincial Key Laboratory of Pathogenic Biology and Epidemiology for Aquatic Economic Animals Ethics Committee.

## Author contributions

SC and JJ designed the experiments. FD completed the experiments and wrote the manuscript under the guidance of SC. SC and JJ revised FD’s manuscript. XW assisted with the fish rearing and injection experiments and GL assisted with the sampling and blood collection. All authors contributed to the article and approved the submitted version.
